# Gut microbiota dynamics and their impact on body condition in nestlings of the yellow-rumped flycatchers, *Ficedula zanthopygia*

**DOI:** 10.3389/fmicb.2025.1595357

**Published:** 2025-05-30

**Authors:** Longru Jin, Pai Zhang, Keping Sun, Haitao Wang

**Affiliations:** ^1^Jilin Engineering Laboratory for Avian Ecology and Conservation Genetics, Northeast Normal University, Changchun, China; ^2^Jilin Provincial Key Laboratory of Animal Resource Conservation and Utilization, Northeast Normal University, Changchun, China; ^3^Jilin Provincial International Cooperation Key Laboratory for Biological Control of Agricultural Pests, Northeast Normal University, Changchun, China; ^4^Key Laboratory of Vegetation Ecology, Ministry of Education, Changchun, China

**Keywords:** gut microbiome, yellow-rumped flycatcher, microbial diversity, nestling, temporal dynamics, SMI

## Abstract

Investigating the gut microbiome during host development is essential for understanding its influence on host health and fitness. While host body condition is a crucial fitness-related trait and a strong predictor of viability in numerous animal species, its relationship with gut bacteria remains underexplored, particularly in non-model organisms. This study examines the gut microbiome of the altricial wild bird species, yellow-rumped flycatchers (*Ficedula zanthopygia*), by analyzing nestling feces through 16S rRNA sequencing at four developmental stages: Day 3, Day 6, Day 9 and Day 12 post-hatching. We explored the temporal dynamics of the gut microbiome and its correlation with body condition, a key indicator of fitness. Our results demonstrate signinficant shifts in microbial community composition and diversity throughout development. Notably, Day 3 nestlings displayed lower alpha diversity compared to later stages, while microbial diversity stabilized from Days 6 to 12. Both the age of the nestlings and the environmental conditions of the nest box significantly shaped the gut microbial community structure. A contemporaneous relationship was observed, where the scaled-mass index (SMI) at Day 6 positively correlating with microbial diversity at that time. Additionally, a time-lagged effect emerged, linking SMI at Day 9 to microbial diversity at Day 6. These findings highlight the vital role of the gut microbiome in the development of nestlings, particularly emphasizing Day 6 as a critical period due to its stable microbial diversity and association with SMI. This study underscores the influence of gut bacteria on host fitness in developing birds.

## Introduction

The gut microbiome plays a fundamental role in host health and fitness (Kasun et al., 2022), especially during early developmental stages (Velando et al., 2021; Diez-Mendez et al., 2023). The early-life establishment of the gut microbiome has been shown to be critical for microbial structure and function later in life (Davidson et al., 2020), influencing key physiological functions, such as digestion and nutrient absorption (Tang et al., 2024), immune system development (Mohamed et al., 2022), and pathogen defense (Yitbarek et al., 2018). Therefore, uncovering the gut microbiome across host development is essential for understanding its role in host health and fitness in natural populations.

Among vertebrates, the establishment of the early-life gut microbiome is particularly important for birds (class Aves), especially for altricial nestlings which spend the brooding period within the nest and are fed directly by their parents. Some key drivers may potentially affect the gut microbiota, such as environment and host traits (Somers et al., 2023). Gut microbiota are sensitive to the environment and can vary in response to external factors such as nesting material and habitat (Goossens et al., 2022; Somers et al., 2023). For altricial species, the gut microbiomes of nestlings tend to be more similar within broods than between broods (Benskin et al., 2015; Teyssier et al., 2018), which may result from the fact that nestmates are fed similar food items by the same parents and raised in the same nest.

Host traits, especially age, have the potential to affect the gut microbiota. The gut microbiota can vary significantly depending on nestling age, and differ across bird species. Some studies have shown that gut bacterial diversity is highest in nestlings and decreases during maturation, as observed in great tits (*Parus major*) (Teyssier et al., 2018; Somers et al., 2023). In contrast, microbial diversity increases gradually with age in species like ostrich (*Struthio camelus*) (Videvall et al., 2019) and shorebirds (Grond et al., 2017), while the short-tailed shearwater (*Ardenna tenuirostris*) maintains relatively stable communities throughout development (Meagan et al., 2017). The conflicting reports on the relationship between host age and gut microbial diversity indicate that summarizing the mechanisms behind the development of gut microbiomes is complex. This also underscores the importance of further research to enhance our understanding of the host gut microbiome across different stages of development.

Variation in gut microbiota in birds is also associated with juvenile traits, such as natal body mass (Beaumont et al., 2016) and amounts of visceral fat (Le Chatelier et al., 2013). For example, a study on wild nestling great tits showed significant shifts in microbiota diversity and composition between Day 8 and Day 15 of development, which were correlated with nestling body condition (Teyssier et al., 2018). It was found that gut microbial diversity at Day 8 can predict nestling weight at Day 15, indicating potential delayed effects on host traits (Davidson et al., 2021). Some studies have also demonstrated that the gut microbiome is an important predictor of host weight, either positively or negatively (Teyssier et al., 2018; Kohl et al., 2019). These findings suggest a relationship between gut microbiota and nestling fitness in birds. However, as limited studies have examined the relationships between gut microbiota and body condition, further research is needed to provide more concrete evidence supporting these hypotheses. Therefore, longitudinal data collection on both the gut microbiome and indicators of fitness, such as body condition, is essential to better understand the impact of the gut microbiota on bird fitness (Rodriguez et al., 2016).

The yellow-rumped flycatcher (*Ficedula zanthopygia*) is a migratory and secondary cavity-nesting bird species, with breeding populations mainly distributed in China, Korea, Mongolia, and Eastern Asian Russia (BirdLife International, 2025). In Northeast China, the breeding ecology of the yellow-rumped flycatcher has been investigated for many years. This species typically builds their nests in early May and begins laying eggs in mid-May (Deng and Zhang, 2016). As an altricial species that readily breeds in artificial nest boxes, the brooding period of *F. zanthopygia* lasts 13.2 days, providing an excellent natural system to track, sample and monitor nestlings throughout the early stages of development in the wild from a nestbox population. Because the neonatal stage is a critical time for gut microbiome colonization in many species (Koenig et al., 2011), we investigated the natural variation of gut microbiome at four development stages from Days 3 to 12 of development before fledging, in order to reveal the substantial shifts and potential influencing factors, such as host age and nest in gut microbiota for nestling yellow-rumped flycatchers. Considering that early developmental windows were important for microbial influences on host traits (Hansen et al., 2012; Davidson et al., 2021), we measured host body condition as an important fitness-related trait, to test whether gut microbial diversity was associated with nestling body condition. We proposed that if diversity is positively related to measures of host fitness by contemporary or time-lagged effects, then a rich microbial diversity would be very important for nestling health development. We expect to find a critical stage when the gut microbiota may have implications for host fitness.

## Materials and methods

### Study area

The study was conducted in the Zuojia Nature Reserve, located in Jilin Province, Northeast China (44°1′–45°0′ N, 126°0′–126°8′ E). The reserve covers an area of 60 km^2^ and has an elevation range of 200–554.6 m. The region experiences a continental monsoon climate typical of the temperate zone. The vegetation is primarily composed of a natural secondary broad-leaved forest, which includes species such as *Betula dahurica*, *Fraxinus mandschurica*, *Quercus mongolicus*, and *Tilia mandshurica*. To attract the yellow-rumped flycatchers and other secondary cavity-nesting bird species (e.g., *Parus minor*), artificial nest boxes made of wood (dimensions: 12 × 12 × 25 cm internally, with a hole diameter of 4.5 cm) were randomly mounted on trees at a height of approximately 2.5–3 m above the ground, with a minimum spacing of 30 m between each box. A total of around 450 nest boxes were maintained year-round.

### Sample collection

The artificial nest boxes were first monitored every 3 days from May to June 2022. The first egg laying dates and brood sizes of yellow-rumped flycatcher were recorded for each nest. As the clutches approached the estimated hatching date, the nest boxes were checked daily. Yellow-rumped flycatchers usually lay 5–6 eggs and the female incubates the eggs for 2 weeks. After hatching, between 10:00 and 15:00, when the weather was warmer, we gently stimulated the cloaca of nestlings with clean latex gloves to encourage defecation. Nestlings from which fecal samples were collected were individually marked with a unique combination of color markings using non-toxic felt pens, and these markings were refreshed with each fecal sample collection. All collected fecal samples were stored in 2 mL sterilized microtubes, placed in an ice box within 2 h of collection, and then stored in a −80°C freezer upon return. We collected fecal samples from the same nestlings at the following ages: Days 3, 6, 9, and 12, whenever possible, as the nestlings fledge approximately 14 days after hatching. However, those that did not defecate within 10 minutes were excluded from the study. In total, 132 fecal samples from 35 nestlings from 7 nests were collected for 16S rRNA gene sequencing (Table 1; Supplementary Table S1). In addition, during each sampling event, the nestlings’ body mass (g) was measured using a digital scale, and tarsus length was measured with a digital caliper as the distance from the tibiotarsus joint to the far end of the last leg scale where the toes emerge (Sonya et al., 2007). Body mass and tarsus length were used to calculate the scaled-mass index (SMI), an indicator of host body condition. SMI was calculated using the equation from the linear regression of log-body mass on log-tarsus length estimated by type-2 (standardized major axis) regression (Peig and Green, 2009). In addition, to reduce the possibility of nest abandonment by parental birds and to avoid nestlings leaving the nest early due to perceived threats from our interference, we collected blood samples (approximately 20 μL per sample) when the nestlings were 9 days old. We wore sterilized gloves to handle the nestlings and gently punctured the brachial vein, using microhematocrit capillary tubes to collect the blood samples. The samples were then stored in absolute ethanol in a −80°C freezer until DNA extraction. The DNA extracted from the blood samples was used for sex identification.

**TABLE 1:** The number of collecting samples of nestling yellow-rumped flycatchers from different nest boxes at four different ages.

Nest ID	Age of nestlings			
	**Day 3**	**Day 6**	**Day 9**	**Day 12**
352	4	4	4	4
21-4-3	5	6	6	6
21-4-5	5	5	5	5
21-A5	6	5	6	1
16-B2	6	6	6	6
16-C0	5	5	5	4
20-E7	3	3	3	3
Total	34	34	35	29

### DNA isolation, library preparation and amplicon sequencing

DNA was extracted from each fecal sample using E.Z.N.A.^®^ soil DNA Kit (Omega Bio-Tek, Norcross, GA, United States) following the manufacturer’s instructions. The concentration and purity of the DNA were determined using a NanoDrop 2000 UV-vis spectrophotometer (Thermo Scientific, Wilmington, United States). Subsequently, the V3-V4 region of the 16S rRNA gene was amplified with universal primers 338F (5′-ACTCCTACGGGAGGCAGCAG-3′) and 806R (5′-GGACTACHVGGGTWTCTAAT-3′). The PCR cycling conditions were as follows: an initial denaturation at 95°C for 3 min, followed by 27 cycles of denaturing at 95°C for 30 s, annealing at 55°C for 30 s, and extension at 72°C for 45 s, and single extension at 72°C for 10 min. The PCR mixtures consisted of 5 × TransStart FastPfu buffer 4 μL, 2.5 mM dNTPs 2 μL, forward primer (5 μM) 0.8 μL, reverse primer (5 μM) 0.8 μL, TransStart FastPfu DNA Polymerase 0.4 μL, template DNA 10 ng, and finally ddH_2_O up to 20 μL. PCR reactions were performed in triplicate. The PCR products from each sample were quantified, pooled and subsequently sequenced by Illumina Novaseq (2 × 250 bp) in Shanghai Majorbio Bio-pharm Technology Co., Ltd. (Shanghai, China).

Furthermore, DNA was extracted from each blood sample using the UNIQ-10 column animal genomic DNA isolation kits (Sangon Biotech, Shanghai, China). We utilized the primer pairs sex1 (5′-CTCCCAAGGATGAGAAACTGTGCAAAACAGGTA-3′) and sex-mix (5′-CCTTCRCTKCCATTRAAGCTRATCTGGAAT-3′) to amplify the chromo helicase DNA binding (CHD) gene for nestling sex identification. The PCR reaction mixture included 10 × PCR buffer, 0.2 mM of each primer, 1.6 mM dNTP mix, 0.4 unit of Taq DNA polymerase, and 0.1 μg of gDNA. Amplification included an initial incubation at 95°C for 8 min, followed by 35 cycles at 94°C for 30 s, 50°C for 45 s, and 72°C for 40 s, with a final extension at 72°C for 8 min. The PCR products were then run on a 2% agarose gel to examine the amplified bands.

### Bioinformatics

The demultiplexed Illumina sequence data was initially merged using FLASH v1.2.11 with a minimum window quality score of 20. Only overlapping sequences longer than 10 bp were assembled based on their overlapped sequence, with a maximum mismatch ratio of 0.2. Samples were distinguished based on the barcode and primers used. Subsequently, we employed the DADA2 pipeline (v. 1.24.0; Callahan et al., 2016) within Qiime2 to process the raw sequencing data. This involved filtering the reads based on quality, merging the paired-end reads, defining unique DNA sequence with 100% sequence identity, generating amplicon sequence variants (henceforward ASV), and constructing a table of ASVs. To remove potentially artifactual sequences, we further filtered the ASV table, retaining only those with at least 0.001% relative abundance in the dataset. Taxonomic classification of each ASV was performed using RDP’s Naive Bayes Classifier (Wang et al., 2007) against the Silva reference database (version 132; Quast et al., 2013). ASVs were excluded if they were not classified as bacteria, not assigned to a specific phylum (as these were considered spurious), or if they were classified as mitochondria or chloroplasts. For all samples, only one sample (sample id: Y352D324, belonging to Nest ID 352, Supplementary Table S1) retained only 6079 reads, with other having more than 14409 reads. Therefore, sample Y352D324 was excluded from further analyses.

We used the “qiime diversity core-metrics-phylogenetic” command in Qiime 2 to compute the alpha diversity indices (Shannon and Chao1 diversity indices) and beta diversity metrics (unweighted UniFrac and Bray-Curtis distances) for the nestling gut microbiome. The “-i-phylogeny” option was included to generate a rooted phylogenetic tree of observed ASVs. We rarefied sequence reads 14,409, with uniform depth of coverage. Bacterial relative abundances were summarized at both the phylum and genus levels. To visualize the shared and unique ASVs across ages, regardless of their relative abundance, Venn diagrams were constructed based on the relative abundance for all phyla and genera using the VennDiagram package (version 1.7.3) of R (v. 4.11.0; R Core Team, 2021).

### Statistical analysis

The following analyses were conducted on one of five subsets of the data. The initial subset, known as the “all birds subset,” consisted of nestlings across all developmental ages [3-day-old nestlings (D3); 6-day-old nestlings (D6); 9-day-old nestlings (D9); 12-day-old nestlings (D12)]. This subset was utilized to explore the relationship between gut microbiota composition and factors including developmental age, sex, first-egg laying date (henceforward laying date) and brood size of nest. Subsequent subsets, from the second to fifth, focused exclusively on nestlings at D3, D6, D9 and D12, respectively. All statistical analyses were carried out using R (v. 4.11.0; R Core Team, 2021) unless stated otherwise.

Linear mixed models (LMM) were used to assess the influence of host traits on two alpha diversity indices: Shannon PD and Chao1 diversity. These models were fitted using the lme4 package (Bates et al., 2015) for each data subset. Significance was determined utilizing Satterwaite’s degrees of freedom method (Satterthwaite, 1946), implemented through the lmerTest package (Kuznetsova et al., 2017). Evaluation of residuals from all models were conducted using DHARMa (Hartig, 2024). DHARMa simulates data based on the provided model and offers a more robust validation approach than simple residual versus fitted data plots for mixed effects models. The suitability of the response variable was verified based on the normality of the model residuals.

In the global models encompassing all nestlings, the following variables were included as main effects: age (D3, D6, D9, D12), sex, brood size of nest, and laying date of the nest. To control for non-independence, the LMM incorporated nested random intercepts for Nest ID and Individual ID. Furthermore, a Bayesian linear mixed-effects model (BLMM) was fitted using the Stan computational framework with the R package brms (v2.18.0) (Bürkner, 2017) to examine which factor best explain variation in alpha diversity (Jones et al., 2023). In this Bayesian regression analysis, the Shannon or Chao1 indices was used as the response variable, and the explanatory variables were the same to those in the LMM. The model incorporated random intercepts for Nest ID and random slopes for Individual ID. Four chains, each with 2000 iterations, were used. In addition, differences in community alpha diversity among different nestling ages were assessed using Wilcox tests.

In order to investigate the potential implications of the gut microbiome on host fitness, we assessed the relationship between the gut microbiome and SMI as a crucial indicator of body condition. Initially, we selected SMI as the response variable, and examined whether nestling SMI was influenced by alpha diversity (Shannon or Chao1), sex, laying date and brood size of each nest at each developmental age, considering them as fixed effects. Nest ID was incorporated as nested random effect in the analysis. In order to avoid quantile deviations or singular fit warnings in DHARMa residual, we obtained the log10 value of some variables. If a significant correlation between alpha diversity and SMI was observed at a specific age period, we could further analyze the correlations between alpha diversity and SMI at other ages using lmer package, incorporating Nest ID as a nested random effect. If singular fit warnings were detected, a linear model would then be used. This analysis specifically focused on nestlings with repeat collected samples to ensure data consistency. We evaluated the correlations between alpha diversity and SMI by calculating Pearson’s correlation coefficient with the “cor.test” function in R.

To examine the bacterial community structures (beta diversity), we employed unweighted UniFrac and Bray-Curtis distances and visualized them using principal coordinate analysis (PCoA) plots. It is worth noting that while both distances evaluate count-based data, unweighted UniFrac assesses presence-absence, whereas Bray-Curtis evaluates relative abundances. To determine the impact of age, sex, and nest on beta diversity, we utilized permutational multivariate analysis of variance (PERMANOVA) through the Qiime 2 command “qiime diversity adonis.” Additionally, we conducted Wilcox tests to investigate microbial community differences between and within nest boxes based on unweighted UniFrac and Bray-Curtis distances.

## Results

### Sequence statistics

After quality processing, removal of chimeras and filtering, we obtained a total of 7,988,246 high-quality reads from 131 fecal samples. The number of reads per sample ranged from 14,409 to 108,612, with a mean of 60,979 reads. These high-quality reads clustered into 3,412 ASVs.

**FIGURE 1:**
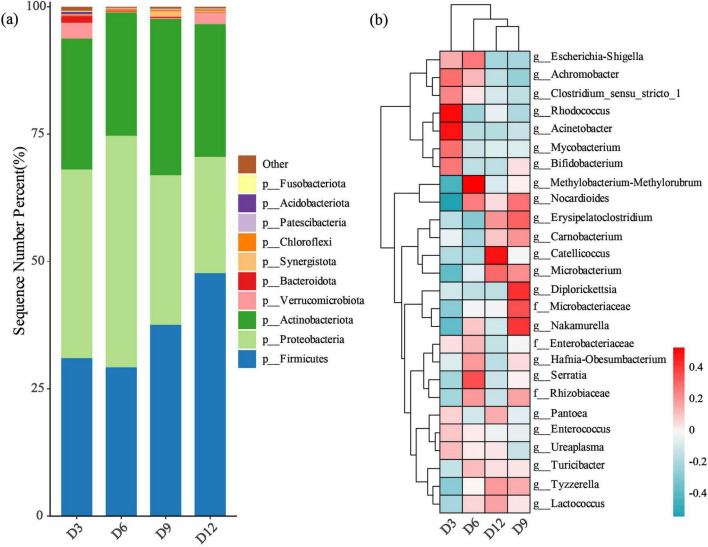
Gut microbial composition in nestling yellow-rumped flycatcher fecal samples. **(a)** The relative abundance of the top 10 bacterial phyla. **(b)** The heatmap of the relative abundance of 26 bacterial genera (> 1% mean relative abundance). Each column represents fecal samples from each age stage. All other taxa are collapsed into the others category.

Across all samples of the four age periods, we identified a total of 35 bacterial phyla. The dominant phyla in terms of mean percentage relative abundance were Firmicutes (36.37%), Proteobacteria (33.68%) and Actinobacteriota (26.65%) (Figure 1a). Among the identified genera, 26 had a mean relative abundance exceeding 1% (Figure 1b). The dominant bacterial composition at the genus level changed across the four age periods (Figure 1b). The dominant bacterial genera at D3 were noticeably different from those at other ages, while the nestlings at D9 and D12 had more similar bacterial genera compared to other ages (Figure 1b).

### Alpha diversity

**FIGURE 2:**
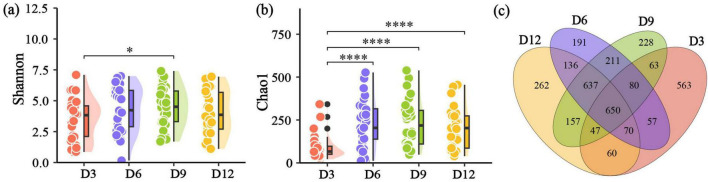
Gut microbial alpha diversity and distribution of ASVs across different nestling ages. **(a)** Shannon diversity. **(b)** Chao1 diversity sampled at different ages. **(c)** Venn diagram showing the overlapping numbers of ASVs between the four different ages in nestling yellow-rumped flycatcher fecal samples. *p < 0.05, **p < 0.01, ***p < 0.001, ****p < 0.0001.

The Shannon diversity index of nestling gut microbiome exhibited similarity across different age periods, except the significant differences between D3 and D9 (Figure 2a). However, Chao1 diversity index at D3 were significantly lower compared to the other three nestling age stages (Figure 2b). Specifically, at D3, 563 unique ASVs were detected, indicating a higher abundance than in the other age periods, which ranged from 191 to 262 unique ASVs (Figure 2c). Both LMM and BLMM analyses showed that only age significantly affected the Chao1 index or had a positive effect on it (Supplementary Tables S2, S3). Additionally, it was observed that sex, laying date and brood size did no exert a significant influence on alpha diversity (*P* > 0.05, Supplementary Tables S2, S3).

### Beta diversity

**FIGURE 3:**
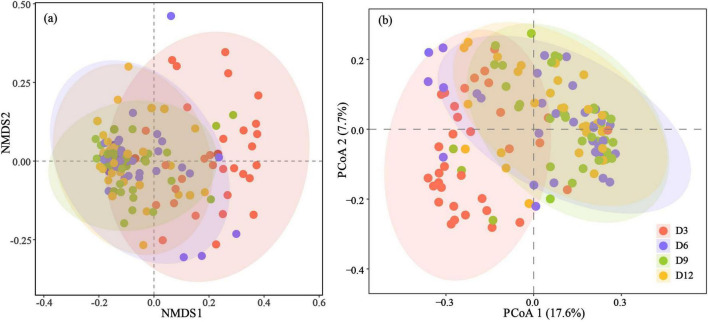
NMDS plot and principal coordinate plot are based on Bray-Curtis distances (stress = 0.213) **(a)** and unweighted UniFrac distances **(b)**. Ellipses represent a 95% confidence interval around the cluster centroids.

Beta diversity varied across age and nests, but not between sexes (Table 2). Significant differences in community composition were observed among early development stages in nestlings, as indicated by both Bray-Curtis distances (PERMANOVA: pseudo-F_4_,_131_ = 2.358, *p* = 0.001) and unweighted UniFrac distances (PERMANOVA: pseudo-F_4_,_132_ = 4.138, *p* = 0.001) (Figure 3). Among the different ages, the microbiota of nestlings at D3 displayed the most substantial differences to all other ages based on both Bray-Curtis distances and unweighted UniFrac distances (Figure 3; Supplementary Table S4). For Bray-Curtis distances, the microbiota at D6 also showed difference with those at D9 (Supplementary Table S4). Furthermore, lower gut microbial variation among nestlings within nests than between nests were observed from D3 to D12 (Supplementary Figure S1).

**TABLE 2:** Influence of age, nest and sex on the composition of gut microbial communities in the yellow-rumped flycatcher nestlings based on adonis tests.

Distance matrix	Variable	SS	*F*_df_	*R* ^2^	*p*
Unweighted UniFrac	Age	2.726	4.365_3_	0.089	0.001*
	Nest	2.677	2.144_6_	0.088	0.001*
	Sex	0.230	1.106_1_	0.008	0.253
Bray-Curtis	Age	2.791	2.515_3_	0.053	0.001*
	Nest	5.181	2.334_6_	0.098	0.001*
	Sex	0.538	1.455_1_	0.010	0.052

### Gut bacteria and nestling body condition

**FIGURE 4:**
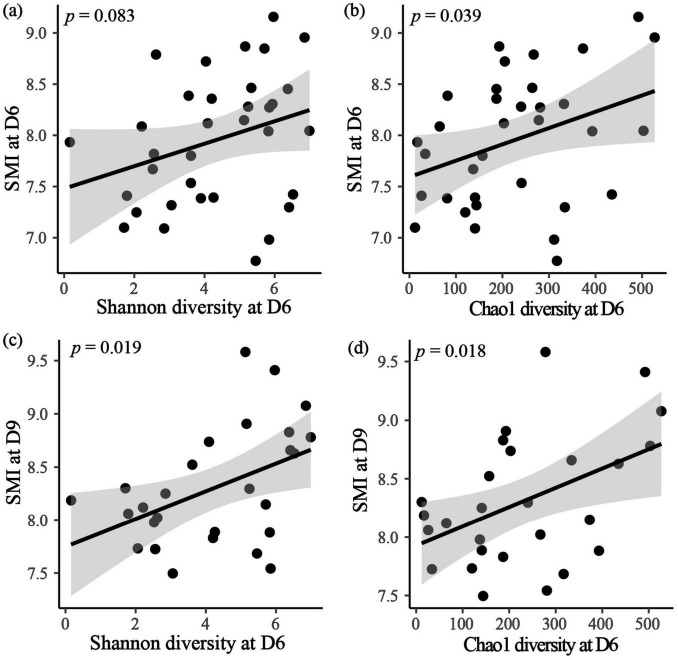
The effect of alpha diversity at D6 on contemporary SMI at D6 **(a,b)**, and time-lagged SMI at D9 **(c,d)**. Black dots are individual data points, black line is the regression line with 95% CI (shaded gray).

In the contemporary analysis, it was found that only SMI at D6 showed positive correlation with Chao1 diversity index at D6 (Figure 4; Table 3; Supplementary Table S5). Furthermore, the time-lagged analysis revealed a positive relationship between SMI at D9 and Shannon diversity as well as Chao1 diversity indices at D6 (Figure 4; Supplementary Table S6). However, no correlation was identified between SMI and alpha diversity at D3 and D12 (Supplementary Table S5).

**TABLE 3:** LMM output from contemporary (day-6) body condition (SMI) analyses.

Dependent/Independent variable	Estimate	*SE*	*df*	Test statistic	*p*
**(a) SMI (day-6)**					
(Intercept)	5.770	1.328	4.994	4.364	0.007*
Shannon	0.123	0.061	28.894	2.022	0.053
Sex	−0.220	0.200	28.781	−1.101	0.280
Laying date	−0.016	0.024	3.595	−0.669	0.544
Brood size	0.309	0.183	3.803	1.682	0.171
**(b) SMI (day-6)**					
(Intercept)	4.486	1.355	7.419	3.577	0.008*
Log10(Chao1)	0.615	0.240	28.959	2.568	0.016*
Sex	−0.281	0.188	28.976	−1.490	0.147
Laying date	−0.016	0.022	3.618	−0.743	0.503
Brood size	0.328	0.166	3.891	1.977	0.121

Nest ID was as random effect. The effect of SMI is reported for alpha diversity of gut microbiota (Shannon and Chao1), sex, laying date and brood size of each nest.

*p < 0.05.

## Discussion

### Characteristic of gut microbioa in the yellow-rumped flycatcher

In this study, the taxonomic composition of nestling yellow-rumped flycatchers’ fecal microbial community was dominated by bacterial from the phyla Firmicutes and Proteobacteria, followed by Actinobacteria. The phylum Firmicutes, which was the most prevalent in nestling yellow-rumped flycatchers, is known to aid in carbohydrate degradation and produce energy-rich short-chain fatty acids which can be absorbed by the host gut, increasing nutrient uptake, weight gain and fat storage in birds (Teyssier et al., 2018; Skeen et al., 2021). Therefore, the prevalence of Firmicutes may assist nestlings in maximizing energy harvesting for their growth. In particular, the nestlings at D12 have much higher relative abundance compared with those at D3-D9, similar the gut microbiota of altricial barn swallow (*Hirundo rustica*) chicks with average 45% Firmicutes (Kreisinger et al., 2017). In yellow-rumped flycatchers, the nestlings at D12 would be close to fledging, so the prevalence of Firmicutes could be higher, as observed in precocial birds that require rapid development and weight gain (Grond et al., 2017). Proteobacteria was the second most abundant phylum within the nestling gut in this study, which is common and abundant in insectivorous birds (Grond et al., 2018; Skeen et al., 2021). This is consistent with the diet of the yellow-rumped flycatcher, which mainly feed on insects. The phylum Actinobacteria, the third most abundant phylum in nestling yellow-rumped flycatchers, is ubiquitous in aquatic and terrestrial ecosystems. They produce secondary metabolites, such as enzymes and antibiotics, which can affect animal health or disease (Cundliffe, 2006).

In addition, Bacteroidota is also a common phylum in most birds (Grond et al., 2018; Capunitan et al., 2020; Tang et al., 2023). However, the nestling yellow-rumped flycatchers were found to have a very low abundance of Bacteroidota (0.45%), which is consistent with several other birds, such as Darwin’s finches (Michel et al., 2018), Eurasian kestrels (*Falco tinnunculus*) (Zhou et al., 2020) and Sichuan partridges (A*rborophila rufipectus*) (Tang et al., 2023). Members of the Bacteroidota phylum are known for their ability to break down a wide variety of polysaccharides, including carbohydrates and plant cell wall components, in the gastrointestinal tracts of vertebrates (Foley et al., 2016). The low proportions of Bacteroidota in the gut microbiota of yellow-rumped flycatchers could be attributed to their insectivorous diet, which is high in dietary protein intake rather than fiber-rich plant components.

### Nestling age impacts gut microbial diversity

The gut microbiome of 3-day-old yellow-rumped flycatchers was found to be highly differentiated from that of subsequent ages, exhibiting lower alpha diversity and a more unique microbial composition. Low gut microbial alpha diversity is common in multiple disease states, while high alpha diversity typically indicative of greater vertebrate host health (reviewed by Heiman and Greenway, 2016). For 3-day-old nestlings, their immunity system and overall health may not be as developed as in older individuals. Actually, this pattern of lows followed by highs mirrors findings in some avian host species. For instance, chickens (*Gallus gallus*) exhibited relatively low alpha diversity indices from days 1 to 7, which then increased and stabilized from days 14 to 28 post-hatch (Awad et al., 2016). Similarly, ostriches showed an increase in alpha diversity after the first week post-hatch, followed by stable gut microbiota from 2 to 12 weeks (Videvall et al., 2019). However, some studies have shown inconsistent patterns compared to our study. For example, in certain avian host species such as arctic shorebirds (0–10 days, Grond et al., 2017) and great tits (day 8 and day 15, Teyssier et al., 2018), the opposite pattern was observed, with a decrease in gut microbial alpha diversity over time. Additionally, in species like the Eurasian kestrel (days 1–25, Zhou et al., 2020), American kestrel (days 5–20, Houtz et al., 2023) and house sparrow (days 3–12, Kohl et al., 2019), the alpha diversity did not vary with age. These findings highlight that the variability in gut microbial dynamics may be attributed to differences in diet or other life history characteristics among different avian species during early development.

In this study, major compositional changes in the gut microbiota occurred during development, especially from the 3-day-old to 6-day-old stage, which could be attributed to the dietary switch from yolk to insects. During the initial 3 days after hatching, yellow-rumped flycatcher nestlings may rely on their internal yolk sac for nutrition. The yolk sac serves as a concentrated source of energy and essential nutrients during the early stages of development (Kuzmina, 2023). After this period, the yolk is mostly absorbed, and the nestlings transition to external food sources, such as insects provided by adult birds. These insects offer a diverse array of proteins, fats, and micronutrients that support rapid growth. Furthermore, the immune system plays a key role in shaping the gut microbiota in birds (Sun et al., 2022), and the relationship between the immune system and gut microbiota during nestling development warrants further investigation to better understand its impact.

In addition, the nest effect significantly changed the gut microbial composition in yellow-rumped flycatcher nestlings. This study observed lower gut microbial variation among nestlings within nests than between nests from D3 to D12. In fact, for altricial birds, gut microbiomes tend to be more similar within broods than between broods (Teyssier et al., 2018; Davidson et al., 2021), which could be influenced by mutually non-exclusive vertical transmission of microbiota from parents during feeding (Chen et al., 2020; Dion-Phenix et al., 2021) and environmental transfer of microbiomes from food and the nest (Jacob et al., 2015; Goodenough et al., 2017; Devaynes et al., 2018). However, microbial alpha and beta diversity did not differ between the sexes in yellow-rumped flycatcher nestlings. Sex-related differences in gut microbial composition may not be apparent until adulthood, when immunosuppressant hormones related to sex are fully developed (Grond et al., 2018).

### Implications for host fitness

In this study, several interesting positive relationships were observed between gut microbial diversity and nestling body condition (SMI) on Day 6 in the yellow-rumped flycatcher, indicating a contemporary effect of the gut microbiota on host traits. This positive relationship is similar to the findings in great tit nestlings (Teyssier et al., 2018). For nestlings, a more diverse gut microbiota could confer greater resistance to pathogen invasions and, in general, greater resilience following perturbations (Buffie and Pamer, 2013). Furthermore, the gut microbial diversity on Day 6 was associated with the SMI on Day 9, suggesting a time-lagged association between the gut microbiota and host traits. A similar time-lagged effect was also observed in great tits, where alpha diversity on D8 was associated with future weight on D15 (Davidson et al., 2020), suggesting that the gut microbiome serves as a mechanism for adaptive phenotypic change through its sensitivity to environmental variability.

## Conclusion

In conclusion, this study revealed dynamic shifts in the gut microbiome of yellow-rumped flycatcher nestlings during early developmental stages, with age and nest-box conditions identified as key drivers of microbial community composition. The stabilization of microbial diversity from Day 6 onward, along with its contemporaneous and time-lagged associations with the SMI, underscores the critical role of gut bacteria in shaping host fitness during development. These findings improve our understanding of microbiome-host interactions in altricial birds and emphasize the importance of temporal and environmental factors in influencing developmental fitness in wild avian species.

## Data Availability

The original contributions presented in the study are publicly available. The 16S rRNA amplicon sequence data can be found at: https://www.ncbi.nlm.nih.gov/, accession number: PRJNA1157539.
