# Supplementary material for: Gut microbiota dynamics and their impact on body condition in nestlings of the yellow-rumped flycatchers, Ficedula zanthopygia
Source: Front Microbiol. 2025 May 30;16:1595357. doi: 10.3389/fmicb.2025.1595357 (PMC12165319; doi:10.3389/fmicb.2025.1595357)
Supplement: Supplementary file 2 [file Data_Sheet_1.docx]

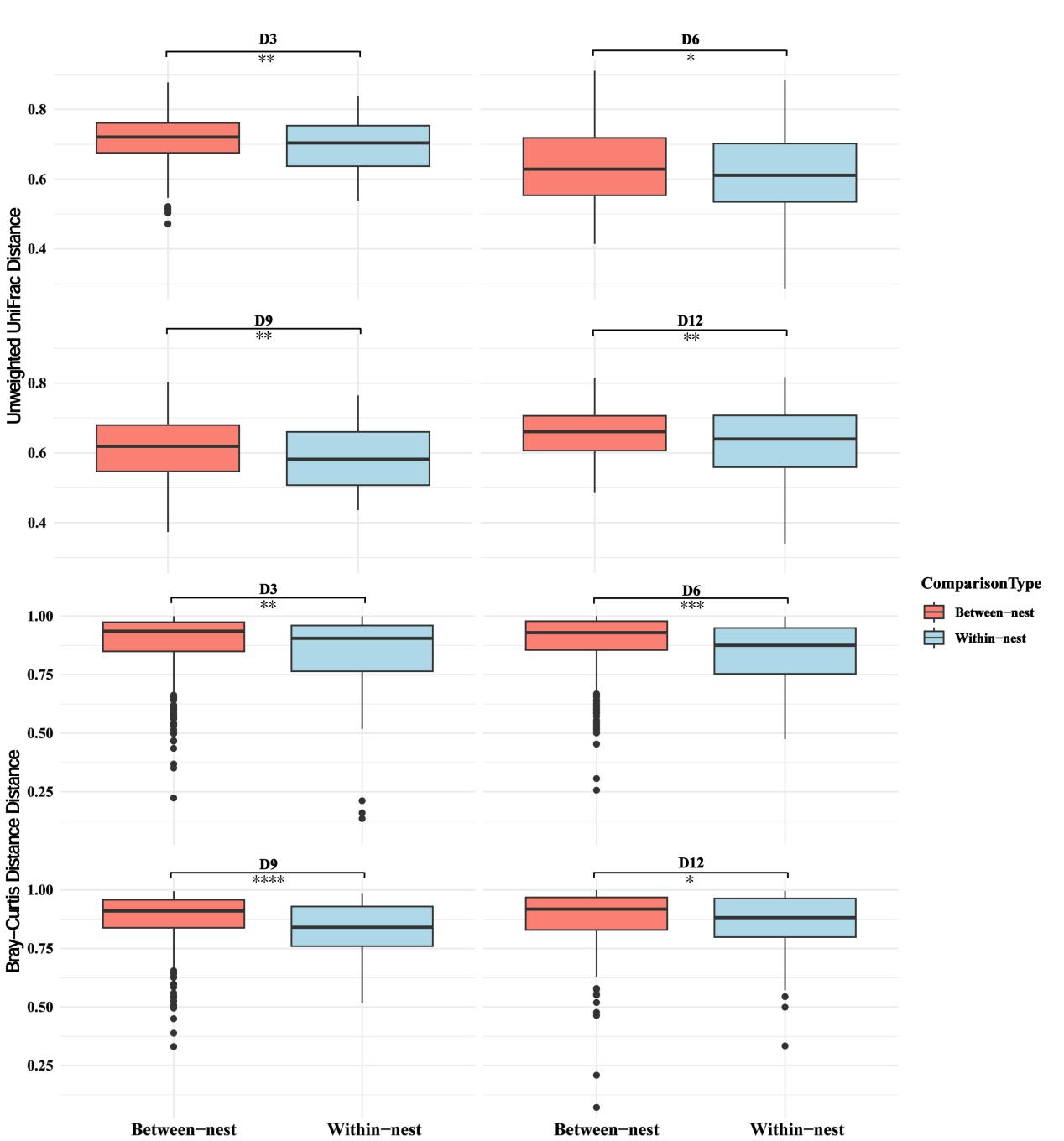


Fig S1 Gut microbial community differences of nestling within and between nests at each nestling developmental ages based on Bray-Curtis distances (a, b, c, d) and unweighted UniFrac distances (e, f, g, h). Differences at D3 (a, e), D6 (b, f), D9 (c, g), and D12 (d, h). *p < 0.05. **p < 0.01. ***p < 0.001. ****p < 0.0001.
